# Evaluation of the Functional Properties and Edible Safety of Concocted Xanthii Fructus Protein

**DOI:** 10.3390/foods14111913

**Published:** 2025-05-28

**Authors:** Yuchen Dong, Zihao Wan, Fuguo Han, Xuemei Fan, Yanli Hao, Fang Wei, Qingfei Liu

**Affiliations:** 1 School of Pharmacy, Shaanxi University of Chinese Medicine, Xianyang 712046, China; dongyc2025@163.com (Y.D.); wanzihao2023@163.com (Z.W.); 2 School of Pharmaceutical Sciences, Tsinghua University, Beijing 100084, China; hanfg2001@163.com (F.H.); xuemeifan@tsinghua.edu.cn (X.F.); haoyanli@tsinghua.edu.cn (Y.H.); weifang0802@126.com (F.W.)

**Keywords:** Xanthii Fructus protein, concocting process, physicochemical characteristics, gut microbiota, untargeted metabolomics, food source

## Abstract

Xanthii Fructus (XF) not only has medicinal function in traditional Chinese medicine (TCM) but also contains rich oil and protein. The aim of this research was to develop the edible value of its protein based on the investigation on the extraction, basic characteristics and functions, safety, gut microbiota, and metabolomics, especially the effect of the concocting process. The proteins from raw and concocted XF were prepared using two methods: alkaline solubilization followed by acid precipitation and ammonium sulfate salting-out, respectively. The secondary structure and physicochemical properties of the proteins were characterized through spectroscopic analysis and property determination. The effects of alkaline and the concocting process on the proteins were systematically compared. The results indicated that the salting-out method could retain the protein activity better. Both alkali treatment and the concocting process altered the folding state of proteins. The toxicological results in mice indicated that a high dose (0.35 g/kg) of raw Xanthii Fructus protein (XFP) might cause damage to the liver and small intestine, and the concocting process could significantly alleviate the damage. The 16S rRNA sequencing technology was used to untangle their impact on gut microbiota in mice and the result showed that raw protein had a certain regulatory effect on Bifidobacterium, Rhodococcus, Lactococcus, and Clostridium, while the concocted protein had a smaller impact, mainly affecting Bacteroides and Bifidobacterium. The untargeted metabolomics using liquid chromatography-mass spectrometry (LC-MS) showed that the proteins of raw XF affected the metabolic level through cysteine and methionine metabolism, purine metabolism, amino sugar and nucleotide sugar metabolism pathways, and the concocted protein mainly involved histidine metabolism and purine metabolism pathways. Overall, XFP had potential development prospects, but the anti-nutritional factors might have some toxicity. The concocting process could significantly improve its safety, and the concocted proteins were worth developing as a food source. In the future, the processing conditions should be further optimized and more systematic investigation should be performed to ensure the safety of XF as a food source.

## 1. Introduction

In recent years, the biological activity and toxicity of plant proteins have gradually garnered significant attention [1]. With increasing health awareness, studies have revealed that the excessive intake of animal proteins may contribute to diseases such as hypertension and hyperlipidemia, driving the popularity of plant-based protein alternatives [2,3,4]. Recent advances in proteomics and biotechnology have enabled substantial progress in plant protein extraction, structural analysis, functional characterization, and bioactivity evaluation [5]. However, challenges persist in their development and utilization [6]. Currently, common plant proteins are primarily derived from legumes and cereals, while other promising sources—such as oilseed crops and non-conventional seeds containing high-quality protein—remain underexplored [7]. Furthermore, standardized protocols for assessing plant protein toxicity remain underdeveloped. Key gaps include the absence of unified allergenicity classification systems and chronic toxicity testing specifications. Different plant proteins exhibit toxicity through distinct mechanisms. For instance, peanuts, although widely consumed, contain the allergenic Ara h 1 protein [8]. Soybeans (used in dairy alternatives) harbor trypsin inhibitors that impair digestion [9]. Croton tiglium seed toxin induces cell membrane damage and cytoplasmic leakage [10]. Ricin from castor beans triggers apoptosis and can be lethal at microgram doses [11,12,13].

Xanthii Fructus (XF), the mature fruit of *Xanthium sibiricum* Patrin ex Widder, demonstrates significant medicinal properties and is rich in oil and protein, indicating the potential for protein-related applications [14]. This annual herbaceous plant is native to temperate and subtropical regions, including East Asia, Europe, and North America. Its fruits are brownish-yellow, obovoid-shaped, and covered with hooked spines. Harvesting typically occurs in autumn at full maturity [15]. Currently, XF oil has been extensively exploited and utilized. Studies indicate that this oil is not only suitable as biodiesel in industrial applications but also contains significant amounts of unsaturated fatty acids with high nutritional value, such as linoleic acid and linolenic acid [16,17]. Among XF components, protein content ranks second only to oil. However, systematic research on the structural functions, biological activities, and food safety of Xanthii Fructus protein (XFP) remains limited. The existing literature primarily reports the presence of the agglutinin protein in raw XF, which is typically consumed after processing in practical applications. Thermal processing, a critical modification method in food manufacturing, is widely recognized for enhancing flavor, improving texture, and reducing protein allergenicity [18,19]. For instance, comparative studies on peanut allergenic proteins demonstrated that boiling and frying alter the secondary structure of Ara h 1, with boiling specifically reducing its allergenicity [20,21]. Solubility—a key functional property influenced by pH, ionic strength, and temperature—exhibits strong correlations with emulsifying and foaming capacities [18]. Highly soluble proteins facilitate both food processing integration and human absorption. Enzymatic hydrolysis of the Prunus japonica protein, for example, increased solubility by 156%, accompanied by 135% and 696% improvements in emulsifying capacity and emulsion stability, respectively [22]. Similarly, quinoa protein solubility reaches near 100% at pH 11 but drops to minimal levels within its isoelectric range (pH 4–6) [23]. The mung bean protein further demonstrates exceptional functional properties, with water/oil holding capacities comparable to commercial soy protein [24]. These findings underscore the necessity for comprehensive investigations into the fundamental characteristics and safety of XFP to advance its development as a food resource. 

The balance of gut microbiota is closely linked to human health, with various dietary components demonstrating targeted regulatory effects [25,26]. Modulating this balance has emerged as a novel strategy for disease prevention and treatment [27]. For example, therapeutic approaches involving gut microbiota regulation have been successfully applied in managing inflammatory bowel disease and type 2 diabetes [28,29,30]. Recent studies have identified persistent differences in gut microbiota composition between moderately malnourished children and their healthy counterparts that remain uncorrected by conventional therapeutic food interventions [31,32,33]. A controlled trial involving 123 malnourished children (aged 12–24 months) revealed that three-month supplementation with microbiota-targeted ready-to-use therapeutic food significantly enhanced physical growth metrics and improved microbial health markers. These outcomes were validated through plasma protein analysis and microbial species quantification [34,35]. Notably, gut microbiota profiles can reflect micronutrient deficiencies [36,37]. Murine models demonstrate that vitamin A deficiency stimulates segmented filamentous bacterial growth [38], whereas deficiencies in vitamins C and E suppress Bacteroides populations [39,40]. These characteristics make gut microbiota an ideal biomarker for evaluating nutritional interventions and food safety profiles [41,42].

Untargeted metabolomics systematically depicts the metabolic profiles of biological tissues, cells, and body fluids, enabling the revelation of metabolic mechanisms through biomarker screening [43]. This methodology has become a vital technical tool in food nutrition, toxicology, and medical research. Common detection techniques include liquid chromatography-mass spectrometry (LC-MS), gas chromatography-mass spectrometry (GC-MS), and Fourier transform infrared spectroscopy (FT-IR) [44]. Notably, untargeted metabolomics demonstrates unique advantages in analyzing complex food systems. For instance, Hydrophilic interaction liquid chromatography-mass spectrometry (HILIC-MS) identified 13 characteristic metabolites generated during coffee bean roasting, serving as roasting markers [45]. Ultra-high performance liquid chromatography-quadrupole time-of-flight tandem mass spectrometry (UHPLC-Q-TOF-MS/MS) detected differential metabolites in tilapia subjected to steaming, boiling, and air-frying treatments [46]. Beyond tracking component changes during food processing, metabolomics also elucidates post-ingestion metabolic mechanisms [47]. A 12-week murine feeding experiment revealed that polar compounds in fried palm oil induced hepatic lesions, glucose tolerance abnormalities, and oxidative damage. Integrated LC-MS and GC-MS analyses identified 36 serum and 18 hepatic differential metabolites in exposed subjects, primarily involving lipid, purine, and amino acid metabolism pathways [48]. Furthermore, gas chromatography-time-of-flight mass spectrometry (GC-TOF-MS) analysis of rat serum demonstrated that thermally processed potato-derived Maillard reaction products altered 13 characteristic metabolites, providing mechanistic insights into their toxicity [49].

Consequently, this study investigates the extraction, characterization, safety evaluation, gut microbiota interactions, and metabolomic profiles of XFP and its processed variants. These findings will establish a scientific foundation for developing the XF protein as a novel edible resource.

## 2. Materials and Methods

### 2.1. Materials

Both raw and processed XF was obtained from Bencaofangyuan Pharmaceutical Co., Ltd. (Beijing, China), and ground prior to use. The concocted XF was prepared through stir-frying according to the relevant standards (article 0213) for Medicinal Processing from the Chinese Pharmacopoeia (2020 edition) [50]. This standardized procedure involves stir-frying dried raw materials until the seed surfaces achieve a yellow-brown coloration. After cooling, the XF spines were removed, and the fruits were sieved for purity control. In this experiment, 5 kg batches of raw and concocted XF were used in separate batches. Following shell removal, the extracted seeds constituted approximately 32.3% of the total mass. The seeds were subsequently ground and sieved through a 3# sieve, yielding approximately 26.2% seed powder.

HPLC-grade methanol, acetonitrile, and formic acid were purchased from Thermo Fisher Scientific (Waltham, MA, USA), while ammonium acetate was acquired from Sigma (Burlington, MA, USA). Deionized water was prepared using a Milli-Q Advantage A10 ultrapure water system (Burlington, MA, USA). All other reagents were of analytical grade.

### 2.2. Preparation of XFP

#### 2.2.1. Alkaline Extraction and Acid Precipitation (AARP/AACP)

An appropriate amount of defatted XF powder (pretreated with petroleum ether) was mixed with 0.05 mol/L sodium hydroxide solution at a 1:80 (*m*/*v*) ratio. The mixture was stirred for 3 h at 400 rpm, then centrifuged at 7100× *g* for 10 min. The supernatant was adjusted to pH 3.8 using 1 mol/L HCl and allowed to stand for 2 h. Subsequent centrifugation under identical conditions yielded protein precipitates. The precipitates were dialyzed through a 1000 Da molecular weight cutoff membrane in deionized water at 4 °C for 24 h, lyophilized, and stored at 4 °C for further analysis.

#### 2.2.2. Salting Method (SRP/SCP)

XF seeds were shelled, pulverized, and sieved before dispersion in phosphate-buffered saline (PBS, pH 7.4) at 1:20 (*m*/*v*). Ultrasonic disruption was performed using a Qsonica LLC instrument (New York, NY, USA) at 20 kHz frequency with 700 W power (100% duty cycle) for 10 min. The homogenate was centrifuged at 18,000× *g* (4 °C) for 20 min. Ammonium sulfate was gradually added to the supernatant at 0 °C until 85% saturation was achieved. After 3 h of ice-water bath agitation at 400 rpm, the solution was recentrifuged under identical conditions. The resultant pellet was reconstituted in PBS (pH 7.4), dialyzed for 24 h at 4 °C, and lyophilized.

### 2.3. Characterization of XFP

#### 2.3.1. XFP Preparation and Electrophoretic Analysis

An appropriate amount of XFP was dissolved in PBS (pH 7.4) to prepare a 15 mg/mL protein solution. The solution was vortexed for homogenization and centrifuged at 7100× *g* for 5 min. The supernatant was collected, mixed with 2× loading buffer, and boiled for 5 min. After boiling, the mixture was centrifuged again under identical conditions and cooled to room temperature for subsequent use. A 10% SDS-PAGE gel was prepared using a commercial kit (Beyotime Biotechnology Co., Ltd., Beijing, China), with stacking and resolving gels optimized for protein separation. Pre-stained protein standards (10–170 kDa, Beyotime Biotechnology Co., Ltd., Shanghai, China) were loaded as molecular weight markers. Protein bands were visualized by Coomassie blue R-250 staining.

#### 2.3.2. Chemical Composition

The protein content in XFP was determined via the Kjeldahl method [51], while moisture content was measured through the direct drying procedure [52]. Ash content was analyzed using high-temperature incineration [53], and fatty acids were extracted via the Soxhlet extraction technique [54]. Carbohydrates were quantified using the phenol-sulfuric acid assay [55].

#### 2.3.3. Amino Acid Composition

To determine the amino acid content in the protein, an appropriate amount of XFP was weighed and mixed with 10 mL of 6 mol/L hydrochloric acid for acid hydrolysis. However, since tryptophan is susceptible to degradation during acid hydrolysis, its content was separately measured using a spectrophotometric method with Ehrlich reagent.

### 2.4. Spectral Structure

#### 2.4.1. Circular Dichroism (CD)

The CD spectra of XFP were recorded using a Chirascan CD spectrometer (Applied Photophysics Ltd., Leatherhead, UK). Protein solutions (0.1 mg/mL) were analyzed in a 1 mm path length quartz cuvette. Ellipticity (mdeg) measurements were performed at 25 °C in continuous scanning mode across 190–260 nm. The average spectrum for each sample was generated from at least three scans using Spectra Manager software.

#### 2.4.2. Fluorescence Spectroscopy

XFP was dissolved in water at pH 8 and pH 12 to prepare test solutions with concentrations of 0.1 mg/mL and 0.5 mg/mL, respectively. Fluorescence spectra were acquired using a Hitachi High-Tech fluorescence spectrometer (Tokyo, Japan) over a wavelength range of 300–500 nm.

#### 2.4.3. Fourier Transform Infrared Spectrum (FTIR)

A mixture of 5 mg XFP and 500 mg KBr was ground in an agate mortar for 10 min, dried at high temperature, and pressed into pellets under compression for 5 min. Spectral scans were recorded between 400 and 4000 cm^−1^.

#### 2.4.4. Ultraviolet Spectrum (UV)

A 0.1 mg/mL XFP solution in pure water was prepared and analyzed using a TU-1900 UV spectrophotometer (Purkinje General Instrument Co., Beijing, China) within the 220–450 nm range.

### 2.5. Functional Properties

#### 2.5.1. Solubility

Following the method of Rezvankhah et al. [56], the solubility of XFP was analyzed across pH values ranging from 2 to 10. Lyophilized powders of AARP, AACP, SRP, and SCP were weighed and dissolved in 50 mL EP tubes containing pure water adjusted to target pH values (using 1 mol/L HCl or NaOH) to prepare 0.5 mg/mL sample solutions. The mixtures were centrifuged at 7100× *g* for 10 min. Protein content in the supernatant was quantified via the Bradford assay, and solubility was calculated using the following formula:

Solubility (%) = P_sup_/P_total_ × 100 (1)

where P_sup_ represents the protein content in the supernatant after centrifugation, and P_total_ denotes the total protein content in the original sample before separation.

#### 2.5.2. Water/Oil Absorption Capacity (WAC/OAC)

Accurately weighed aliquots (0.5 mg/mL) of lyophilized AARP, AACP, SRP, and SCP powders were suspended in pure water within 15 mL EP tubes. After vortex mixing, samples were incubated for 30 min, centrifuged at 7100× *g* for 10 min, and the supernatant was discarded. The precipitated pellet mass was measured. Experiments were performed in triplicate. The WAC was calculated as follows:

WAC (g/g) = (m_2_ − m_1_)/m_1_ (2)

where m_1_ is the dry mass of lyophilized protein powder, and m_2_ represents the mass of the insoluble pellet after centrifugation.

Lyophilized protein powders (0.5 g) were mixed with 3 mL of salad oil in 15 mL EP tubes. Following vortex mixing and 30 min incubation at room temperature, samples were centrifuged at 7100× *g* for 10 min. The oil-saturated pellet mass was recorded. Triplicate measurements were conducted. The OAC was calculated as follows:

OAC (g/g) = (m_oil_ − m_dry_)/m_dry_ (3)

where m_dry_ is the initial dry powder mass, and m_oil_ denotes the oil-bound pellet mass post-centrifugation.

#### 2.5.3. Thermal Stability

Appropriate amounts of AARP, AACP, SRP, and SCP were accurately weighed and placed in a crucible for sealing, then transferred to a differential scanning calorimeter (Mettler Toledo, Greifensee, Switzerland). Thermal stability analysis was conducted over a temperature range of 35 °C to 180 °C at a heating rate of 10 °C/min.

#### 2.5.4. Emulsion Activity Index (EAI) and Emulsion Stability Index (ESI)

Emulsifying properties and stability of XFP at pH 2–10 were determined according to Rezvankhah’s method [56]. A 0.5 mg/mL sample solution was mixed with salad oil (3:1 *v*/*v*) using a high-speed disperser at 10,000 rpm for 2 min. Fifty microliters of the emulsion from the bottom layer were transferred to a 10 mL EP tube containing 5 mL of 0.1% SDS (*w*/*v*). Absorbance values (A_0_) at 500 nm were measured immediately using a UV spectrophotometer (TU-1900, Beijing Purkinje General Instrument Co., Ltd., Beijing, China). After 30 min of standing, absorbance (A_t_) was remeasured. Triplicate experiments were performed. The calculations were as follows:

EAI (m^2^/g) = (2 × 2.303 × A_0_ × DF)/(c × ϕ × 10,000) (4)

ESI (min) = (A_0_ × t)/(A_0_ − A_t_) (5)

where A_0_ represents the absorbance of diluted emulsion at 500 nm immediately after homogenization, DF represents the dilution factor (100), c represents the sample concentration (g/mL), ϕ represents the volume fraction of the oil phase (0.25), and A_t_ represents absorbance after 30 min.

#### 2.5.5. Foaming Capacity (FC) and Foaming Stability (FS)

The FC and FS of XFP at pH 2–10 were analyzed using Rezvankhah’s protocol [56]. A 0.5 mg/mL sample solution was homogenized at 10,000 rpm for 2 min. The initial volume (V_0_) and post-whipping volume (V_1_) were recorded. After 30 min at room temperature, the final volume (V_2_) was measured. Triplicate experiments were conducted. The calculations were as follows:

FC (%) = (V_1_ − V_0_)/V_0_ × 100% (6)

FS (%) = (V_2_ − V_0_)/(V_1_ − V_0_) × 100% (7)

where V_0_, V_1_ and V_2_ represent the pre-homogenization volume, post-homogenization volume (with foam), and post-standing volume, respectively.

### 2.6. Safety Assessment Using Animal Models

#### 2.6.1. Animals and Drug Administration

Seventy-five male ICR mice (18–22 g) were obtained from Vital River Laboratories. The mice were housed at Tsinghua University’s Animal Experiment Center under controlled environmental conditions (22–23 °C, 60–70% humidity) with a 12 h light/dark cycle, following a 7-day acclimatization period. All experimental procedures complied with the ethical guidelines of the Tsinghua University Laboratory Animal Use and Management Committee (Approval Code: THU-LARC-2025-006). Animals were randomly allocated into five groups (n = 15/group): Ctrl: Daily oral gavage with deionized water, RH (high-dose raw herb): 0.35 g·kg^−1^ SRP, RL (low-dose raw herb): 0.02 g·kg^−1^ SRP, CH (high-dose concocted herb) 0.35 g·kg^−1^ SCP, and CL (low-dose concocted herb): 0.02 g·kg^−1^ SCP. Treatments were administered daily for 14 consecutive days.

#### 2.6.2. Sample Collection

Body weights were monitored at 48 h intervals throughout the experimental period. After completing the 14-day treatment regimen, mice were subjected to a 12 h fasting protocol with free access to water prior to specimen collection. Fecal samples (150–200 mg) were immediately flash-frozen in liquid nitrogen and archived at −80 °C for subsequent microbiota profiling. Blood samples acquired through orbital venous plexus puncture were centrifuged at 1400× *g* for 10 min to isolate plasma, with 50 μL aliquots cryopreserved at −80 °C for metabolomic studies and the remainder utilized for ALT/AST biochemical analysis. Systematic tissue processing included the following: glutaraldehyde fixation of jejunal segments for transmission electron microscopy; saline-perfused hepatic and renal tissues that were weighed and preserved in 4% paraformaldehyde for histopathological evaluation; and ileal specimens with luminal contents preserved separately at −80 °C for enzymatic activity assays (ANP and trypsin).

### 2.7. UHPLC-Q-Exactive Orbitrap MS Analysis

#### 2.7.1. UHPLC and HRMS Parameters

Chromatographic separation was achieved using a Thermo Scientific U3000 UHPLC (Waltham, MA, USA) system equipped with a BEH Amide column (2.1 × 100 mm, 1.7 μm particle size). The mobile phase comprised (A) 10 mM ammonium acetate in 0.1% formic acid aqueous solution and (B) 0.1% formic acid in acetonitrile, delivered at 0.3 mL/min with column oven maintained at 35 °C. A multistep gradient program was implemented: 0–5.0 min, 100% B; 5.0–6.0 min, 100–75% B; 6.0–15.0 min, 75% B; 15.0–16.0 min, 75–50% B; 16.0–25.0 min, 50% B; 25.0–26.0 min, 50–100% B; and 26.0–27.0 min, 100% B.

Mass spectrometry data acquisition was performed using a Q Exactive Orbitrap high-resolution mass spectrometer in Full MS-ddMS2 detection mode. Both positive and negative ion modes were scanned separately with a scan range of *m*/*z* 100–1200. The MS1 resolution was set to 70,000, and the MS2 resolution was set to 17,500. The ion source voltage was maintained at 3.2 kV. The capillary temperature was set to 320 °C, and the auxiliary gas heater temperature was kept at 350 °C. The sheath gas flow rate was adjusted to 40 L/min, while the auxiliary gas flow rate was set to 15 L/min. The AGC Target was configured to 1 × 10^6^, and TopN was set to 5. For MS2 scanning, a stepped fragmentation voltage (NCE) was used, with collision energies set at 30, 40, and 50.

#### 2.7.2. Sample Preparation

Ten plasma samples were collected from each of the RL, CL, and Ctrl groups, with each sample containing 50 μL. A pooled “quality control” (QC) sample was prepared by mixing equal aliquots (5 μL) from all prepared plasma samples. Four times the volume of acetonitrile–methanol (1:1) solution was added to each sample, followed by centrifugation at 18,000× *g* for 10 min. The supernatant was then transferred to an EP tube and dried in a freeze dryer. After drying, 100 μL of 50% acetonitrile aqueous solution was added, vortexed for 3 min, and sonicated for 5 min. The samples were then centrifuged again at 18,000× *g* for 10 min, and the supernatant was collected in a sample vial for LC-MS/MS analysis.

### 2.8. Gut Microbiota Sample Processing

DNA was extracted from mouse fecal samples using the OMEGA Soil DNA Kit (Omega Bio-Tek, Inc., Norcross, GA, USA). The V3–V4 hypervariable regions of the bacterial 16S rRNA gene were amplified with primers 338F (5′-ACTCCTACGGGAGGCAGCA-3′) and 806R (5′-GGACTACHVGGGTWTCTAAT-3′). Amplification products were purified with magnetic beads and subsequently quantified via fluorescence-based methods.

### 2.9. Data Processing and Statistical Analyses

Raw metabolomics data underwent analysis through Compound Discoverer 3.2 software (Thermo Fisher Scientific, Waltham, MA, USA), with the workflow comprising peak extraction, alignment, retention time correction, and peak area quantification. Metabolite structural identification was achieved by cross-referencing a local database with the mzCloud online platform.

For gut microbiota analysis, seqduencing libraries were prepared using the Illumina TruSeq Nano DNA LT Library Prep Kit, following manufacturer protocols. Paired-end sequencing was performed on the Illumina NovaSeq platform. Raw sequencing data were processed through the QIIME2 dada2 pipeline and Vsearch software (v2.13.4) for quality filtering, noise reduction, and operational taxonomic unit (OTU) clustering. Subsequent analyses included alpha diversity, beta diversity, and differential abundance testing.

Statistical evaluations employed independent two-sample *t*-tests, with the results expressed as the mean ± SD. Analyses were conducted using IBM SPSS 22.0 (IBM Corp., Armonk, NY, USA), while data visualization was implemented in Origin 2022 (OriginLab Corp., Northampton, MA, USA)

## 3. Results and Discussion

### 3.1. Characterization of XFP

#### 3.1.1. XFP Profile

**Figure 1:**
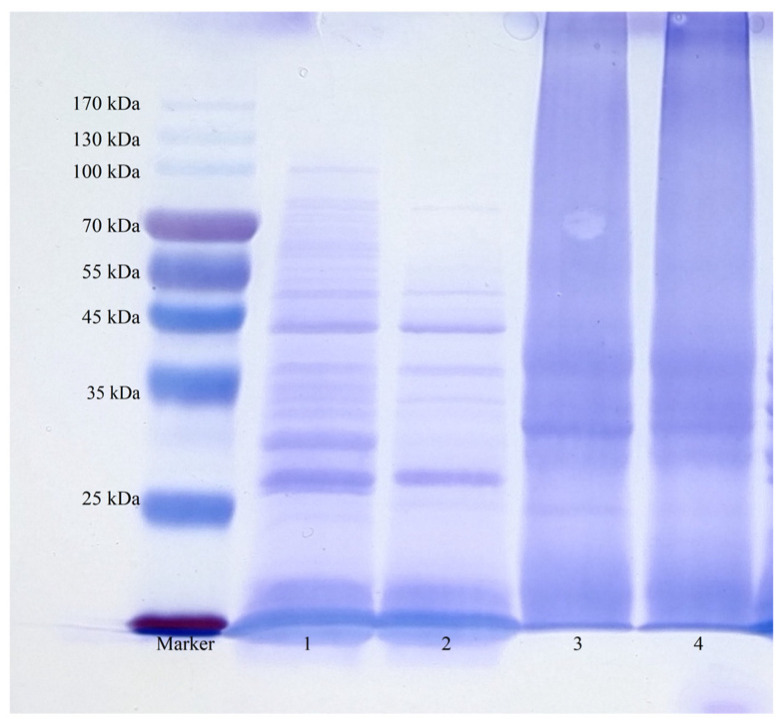
Electrophoretic patterns of XFP extracted using different methods (1, SRP; 2, SCP; 3, AARP; 4, AACP).

The effects of different extraction methods on XFP were analyzed by SDS gel electrophoresis (Figure 1). The crude protein extracted from raw XF using salt fractionation contained multiple subunits with molecular weights primarily distributed between 25 kDa and 55 kDa. In contrast, concocted XF extracts exhibited fewer protein bands (lanes 1–2), likely due to heat-induced denaturation causing intracellular protein coagulation and impaired precipitation. Protein subunits obtained through alkali-soluble acid extraction showed irregular distribution, leading to diffuse electrophoresis bands. Both raw and concocted XFP displayed significant structural degradation (lanes 3–4), consistent with the findings by Tizazu H. Mekonnen [57], where alkaline solutions induced more severe protein degradation than thermal treatment. Comparatively, salt fractionation better preserved native protein structures.

#### 3.1.2. Proximate Analysis of XF Seeds

**Figure 2:**
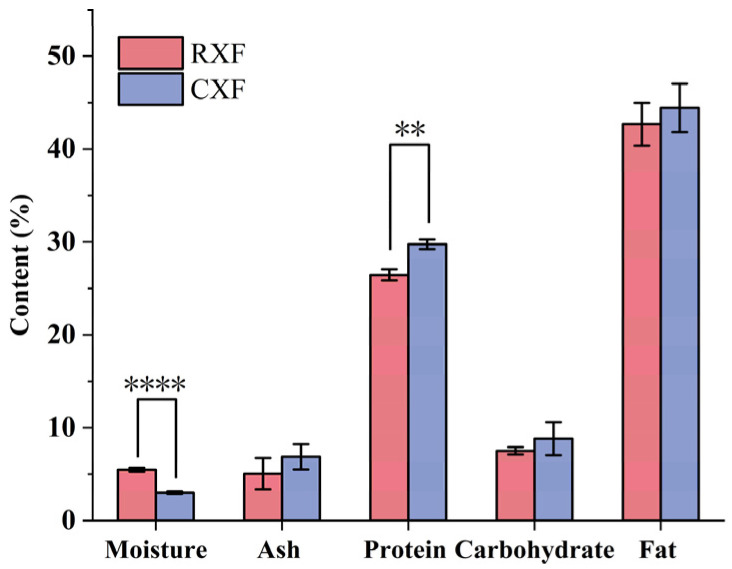
Chemical composition of XFP (mean ± SD, n = 3). ** *p* < 0.01 vs. RXF; **** *p* < 0.0001 vs. RXF.

The proximate composition of XF seeds is shown in Figure 2, including moisture, ash, protein, carbohydrates, and fatty acids. After processing, the moisture content decreased from 5.47 ± 0.22% to 3.01 ± 0.12%, while the protein content increased from 26.46 ± 0.59% to 29.75 ± 0.52%.

#### 3.1.3. Amino Acid Profile of XFP

The nutritional value of protein depends on the diversity, content, and proportion of its amino acids. As shown in Table 1, XFP contains nine essential amino acids: threonine (Thr), valine (Val), methionine (Met), isoleucine (Ile), leucine (Leu), phenylalanine (Phe), lysine (Lys), histidine (His), and tryptophan (Trp). In contrast, the mung bean protein contains only seven essential amino acids, whereas the quinoa protein is unique among cereal proteins in containing all nine, significantly surpassing other plant proteins in nutritional value [58,59]. The Val content in SRP was comparable to that of the quinoa protein, although it exhibited a slight reduction post-processing [60].

Plant proteins often face limitations due to key amino acid deficiencies [61,62]. For example, wheat—a staple in German and Central European diets—lacks sufficient lysine (Lys), an essential amino acid critical for calcium absorption and collagen synthesis [63]. This deficiency may compromise bone health and tissue repair in populations with limited dietary diversity [64,65]. Notably, the Lys content in SRP and SCP was 3.91 ± 0.02 g/100 g and 2.91 ± 0.04 g/100 g, respectively, partially addressing the Lys deficiency in cereal-based proteins.

The biological value of plant proteins is further optimized through the synergistic interaction of non-essential and essential amino acids [61]. Glutamic acid (Glu), one of the most abundant non-essential amino acids, is prevalent in plant proteins such as soybean and wheat, reflecting its role as a key intermediate in plant metabolism. The Glu content in SCP (28.16 ± 0.15 g/100 g) significantly exceeded that in rice (13.9 g/100 g) [66] and approached the highest levels observed in wheat protein (31.7 g/100 g). Glycine (Gly), another non-essential amino acid, supports digestive health through its involvement in glycogenolysis and glucose metabolism. The Gly content in SCP (4.74 ± 0.03 g/100 g) was comparable to that in wheat protein (4.2 g/100 g) [60]. These findings demonstrate that XF, with its rich amino acid profile, can be strategically combined with other plant proteins to achieve optimal nutritional complementarity.

### 3.2. Structural Characteristics of XFP Extracted at Different pHs

#### 3.2.1. CD Spectroscopy

**Figure 3:**
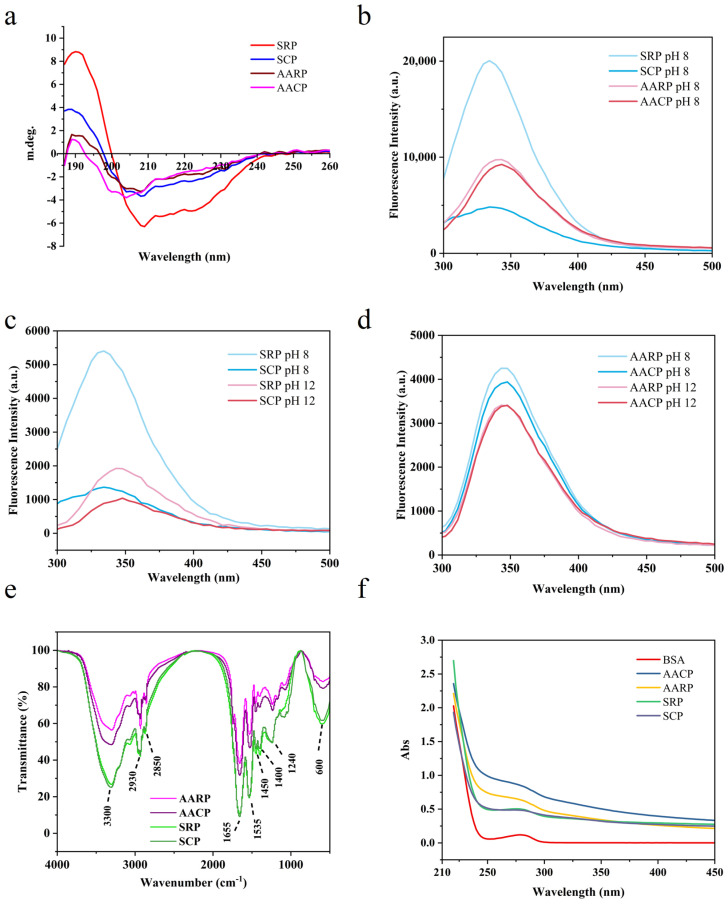
Spectra profiles of XFP: (**a**) CD spectra of XFP; (**b**–**d**) fluorescence spectrum of XFP; (**e**) FTIR spectra of XFP; (**f**) UV spectra of XFP.

Circular Dichroism (CD) spectroscopy revealed distinct secondary structure variations among XFP samples (Figure 3a). The SRP sample exhibited a characteristic α-helix signature [67], with a prominent positive peak at 190 nm, a zero-crossing at 200 nm, and a negative peak at 220 nm. In contrast, SCP displayed reduced peak intensity, likely attributable to partial protein denaturation during cooking processing. This thermal treatment disrupted hydrogen bonding and hydrophobic interactions, resulting in decreased α-helix content, increased random coils, and structural loosening. Notably, AARP and AACP showed minimal characteristic peaks, suggesting complete secondary structure destruction via alkaline treatment [68].

#### 3.2.2. Fluorescence Spectrum

Fluorescence emission in proteins predominantly originates from aromatic amino acids, particularly tryptophan, with tyrosine and phenylalanine contributing to a lesser extent [69]. As shown in Figure 3b–d, the fluorescence spectra of XFP extracted via two distinct methods under varying pH conditions were systematically compared. In Tris-HCl buffer (pH 8.0), the spectral profiles of SRP, SCP, AARP, and AACP revealed significant differences (Figure 3b). Notably, SRP exhibited markedly higher fluorescence intensity than other samples, whereas SCP displayed reduced intensity. This observation suggests that thermal processing altered protein conformation. Figure 3c demonstrates the pH-dependent fluorescence behavior of salt-fractionated proteins. At pH 8 and pH 12, the emission peak underwent a red shift accompanied by decreased intensity, implying partial protein unfolding under alkaline conditions [70]. This structural change likely exposed hydrophobic tryptophan residues to the polar solvent, thereby reducing excited-state energy and inducing a blue shift. For alkaline-acid extracted proteins (Figure 3d), the peak intensity diminished at pH 12 without spectral shifting. This distinct pattern indicates that alkaline pretreatment had already disrupted the native protein architecture, resulting in maximal tryptophan exposure prior to pH adjustment.

#### 3.2.3. FTIR Analysis

Fourier-transform infrared spectroscopy (FTIR) was employed to characterize the secondary structure of proteins. As shown in Figure 3e, the infrared spectra of XFP exhibited characteristic absorption peaks corresponding to hydroxyl and amino group stretching vibrations (3200–3400 cm^−1^), the amide I band (1655 cm^−1^), and the amide II band (1540 cm^−1^) [71]. Notably, SRP and SCP displayed strong absorption in these regions. The amide I band primarily originates from α-helix and β-sheet conformations, whereas the amide II band arises from N–H bending vibrations and C–N stretching vibrations. A significant reduction in absorption peak intensity was observed for AARP and AACP, suggesting a structural disruption of their secondary configurations.

#### 3.2.4. UV Analysis

UV absorption spectra of BSA and XFP were recorded between 220 nm and 450 nm (Figure 3f). Both samples exhibited distinct absorption peaks at 280 nm, confirming the presence of protein components in XFP [72].

### 3.3. Functional Properties of XFP

#### 3.3.1. Solubility of XFP

**Figure 4:**
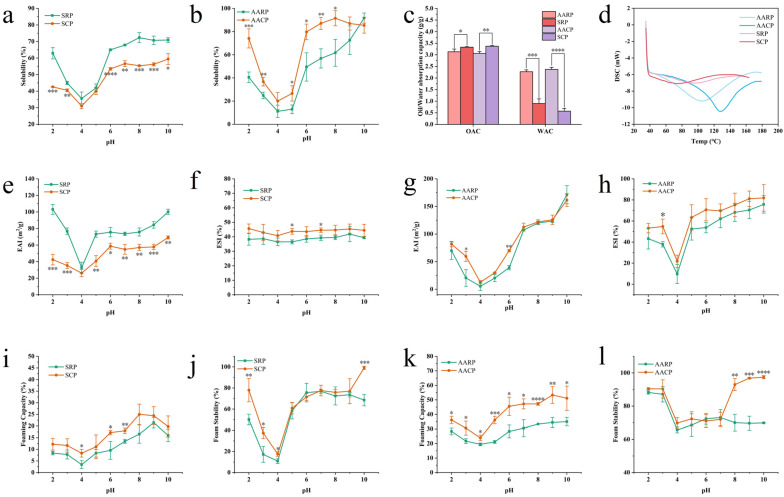
Physicochemical properties of XFP: (**a**) solubility of SRP, SCP; (**b**) solubility of AARP, AACP; (**c**) WAC/OAC of XFP; (**d**) thermal stability of XFP; (**e**) EAI of SRP, SCP; (**f**) ESI of SRP, SCP; (**g**) EAI of AARP, AACP; (**h**) ESI of AARP, AACP; (**i**) FC of SRP, SCP; (**j**) FS of SRP, SCP; (**k**) FC of AARP, AACP; (**l**) FS of AARP, AACP. SRP vs. SCP: * *p* < 0.05, ** *p* < 0.01, *** *p* < 0.001, **** *p* < 0.0001; AARP vs. AACP: * *p* < 0.05, ** *p* < 0.01, *** *p* < 0.001, **** *p* < 0.0001.

Solubility is a critical functional property of proteins, reflecting their capacity to dissolve and disperse uniformly in solution. This property is influenced by factors such as pH, ionic strength, and temperature. As shown in Figure 4a,b, the solubility of XFP decreased significantly within the pH range of 2–4 and increased gradually from pH 4 to 10. The minimum solubility occurred near pH 4, suggesting proximity to XFP’s isoelectric point. Notably, AARP and AACP demonstrated higher solubility than SRP and SCP, likely due to the alkaline-induced disruption of hydrogen bonds, which enhances protein hydrophilicity [73].

#### 3.3.2. WAC/OAC

Water-absorption capacity (WAC) and oil-absorption capacity (OAC) are key functional properties determining protein applicability in food, pharmaceutical, and industrial systems. WAC measures moisture retention within protein matrices, whereas OAC reflects oil-binding ability, which critically impacts food texture and flavor. As illustrated in Figure 4c, alkaline processing did not markedly alter the WAC or OAC of XFP. However, SRP and SCP exhibited higher OAC but lower WAC compared to modified variants. This divergence may stem from their preserved hydrophobic surface structures, which favor oil adsorption, while increased hydrophobic group density limits water molecule interactions. For instance, the modified pea protein’s OAC increased from 2.1 g/g to 3.5 g/g, aligning with SCP values [74]. Similarly, the flaxseed protein’s WAC rose from 1.5 g/g to 3.0 g/g after microwave treatment, attributable to β-conformation conversion to random coils. This mechanism parallels XFP behavior, where the secondary structure is reduced and the random coils’ WAC increased from 0.5 g/g to 2.3 g/g [75].

#### 3.3.3. Thermal Stability of XFP

Differential scanning calorimetry (DSC) is a widely used method for assessing the thermal stability of proteins, which depends on hydrophobic interactions, ionic bonds, disulfide bonds, and aromatic ring interactions within the protein structure. As shown in Figure 4d, the denaturation temperature and enthalpy change in SRP were 98.14 °C and 41.42 J/g, respectively. For SCP, these values decreased to 64.73 °C and 31.32 J/g. Heat treatment significantly reduced the denaturation temperature, promoted structural unfolding, and increased enzyme cleavage site accessibility, thereby enhancing protein digestibility. Notably, alkali-treated proteins exhibited higher thermal stability; AARP had a denaturation temperature of 109.77 °C (ΔH = 137.57 J/g), while AACP reached 125.60 °C (ΔH = 66.30 J/g). In daily life, humans predominantly consume cooked foods. Although alkali treatment elevates protein denaturation temperatures, high-temperature baking may induce the Maillard reaction, generating advanced glycation end products (AGEs) [76] that are potentially harmful to health.

#### 3.3.4. EAI and ESI

The Emulsifying Activity Index (EAI) quantifies a protein’s ability to form stable emulsions at water–oil interfaces, while the Emulsion Stability Index (ESI) measures the emulsion’s capacity to maintain structural integrity over time. As shown in Figure 4e,f, both the SRP and SCP exhibited comparable EAI and ESI patterns. Similarly, AARP and AACP displayed analogous trends in Figure 4g,h. The emulsification profile followed a distinctive “V”-shaped curve, mirroring solubility behavior and reaching minimal values at pH 4. The legume proteins demonstrate superior emulsifying properties, making them ideal candidates for protein-fortified beverage formulations. Notably, heat-treated soy globulin nanoparticles undergo increased particle size and surface hydrophobicity, enhancing emulsion stability through interfacial reinforcement [77]. With an EAI of 58.68 m^2^/g, SCP approached the performance of red bean protein (65.2 m^2^/g), suggesting both proteins hold significant potential for functional food applications [78].

#### 3.3.5. FC and FS

Foaming Capacity (FC) and Foam Stability (FS) are critical parameters for assessing a protein’s ability to form bubbles at gas–liquid interfaces and maintain the structural integrity of foam systems [79]. Figure 4i,j demonstrate the FC and FS of SRP, while Figure 4k,l illustrate these properties for AARP. Numerous food applications, including cakes, ice cream, and beer, require proteins with exceptional FC [80]. Although XFP exhibits significantly higher FC than the soy protein isolate, it remains inferior to whey, quinoa, and wheat proteins [60,81,82,83]. Given FC’s industrial significance, further investigation is warranted to evaluate XFP’s potential as a food-grade foaming agent.

### 3.4. Animal Experiments

#### 3.4.1. Body Weight

Body weight changes in mice from all treatment groups and the Control group were recorded over a 14-day period (Figure S1). The RH group showed a statistically significant reduction in final body weight compared to the Control group (*p* < 0.05), suggesting that SRP exerted toxic effects. However, no significant differences were observed in the low-dose group, indicating the dose-dependent toxicity of SRP.

#### 3.4.2. Organ Index

Organ indices are summarized in Table 2. Neither the liver nor kidney indices differed significantly between treatment groups and the Control group (*p* > 0.05), demonstrating the safety profile of the proteins.

#### 3.4.3. Biochemical Indices

ANP is involved in protein degradation, nutrient absorption, signal regulation, and immune modulation. Trypsin, in contrast, breaks down macromolecular proteins into peptide segments and activates other digestive enzymes [84]. The activities of both enzymes in murine intestinal contents are shown in Figure S2a,b. A significant decrease in enzymatic activity was observed in the RH and RL groups compared to controls (*p* < 0.05), whereas the CL group showed no notable difference. This suggests that intestinal anti-nutritional factors may inhibit enzymatic function. The plasma levels of ALT and AST are presented in Figure S2c,d. The RH group exhibited abnormally low values (*p* < 0.05), while the RL group demonstrated significant improvement, indicating a dose-dependent response. The organ indices (Table 2) revealed no statistically significant differences in the liver or kidney parameters between the treatment and Control groups (*p* > 0.05), confirming the safety profile of the administered proteins.

#### 3.4.4. HE Staining Sections

**Figure 5:**
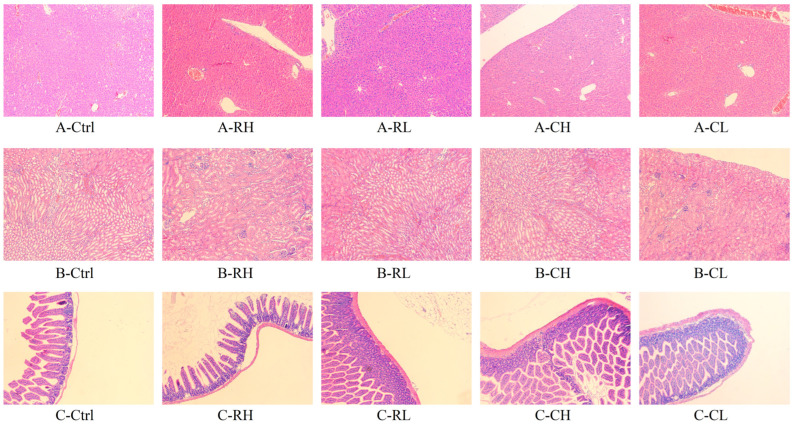
Histopathological examination of mice following administration of RH, RL, CH, and CL ((**A**): liver; (**B**): kidney; (**C**): small intestine) (100×). This is because the light intensity is adjusted when shooting, so that the picture appears in different colors.

Histological sections stained with hematoxylin–eosin (HE) revealed distinct pathological alterations across the experimental groups. Figure 5 illustrates the liver, kidney, and small intestine sections from mice. Compared to the Ctrl group, the RH group displayed hepatocyte necrosis and inflammatory cell infiltration in hepatic tissues. In contrast, both the CH and CL groups retained identifiable hepatic lobule architecture, with hepatocytes arranged regularly around central veins and mild interstitial inflammatory infiltration. The kidney sections showed no significant pathological damage across all groups. In the small intestine, the RH group specimens exhibited partial mucosal damage and irregular villus arrangement. Conversely, the CH and CL groups maintained the structural integrity of intestinal epithelium, with clearly defined submucosal adipose tissue and no apparent inflammation or necrosis.

#### 3.4.5. TEM Observations

**Figure 6:**
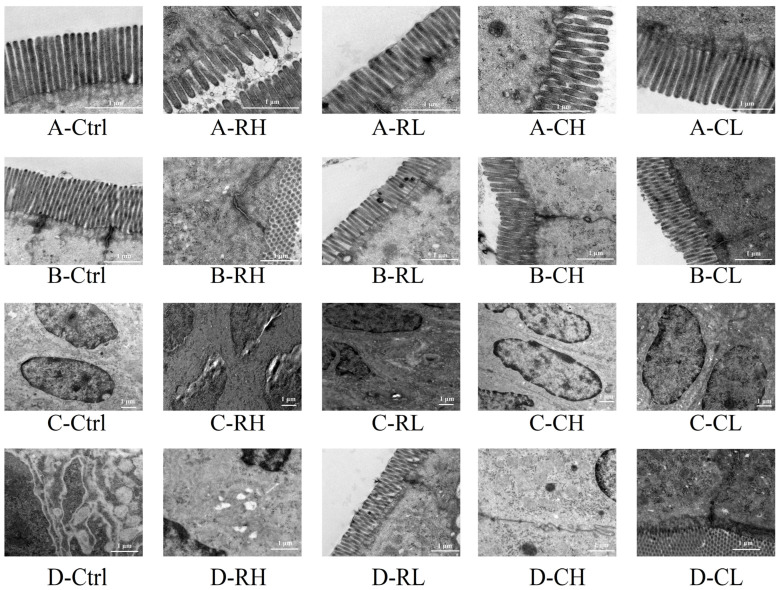
TEM photograph of the jejunum ((**A**): jejunum microvilli; (**B**): intercellular connections; (**C**): cell nuclei; (**D**): cytoplasm).

Transmission electron microscopy (TEM) was employed to examine the jejunal ultrastructure. The results are presented in Figure 6, with quantitative measurements of microvilli length and intercellular connection width provided in Supplementary Table S1. In the Control (Ctrl) group, tightly arranged microvilli, intact intercellular connections, regular nuclear morphology, and the absence of cytoplasmic vacuolation were observed, indicating preserved jejunal barrier function and cellular vitality. In contrast, the RH group exhibited severe structural alterations, including shortened and sparsely distributed microvilli, nuclear membrane rupture, and multiple cytoplasmic vacuolar regions. The RL group displayed milder damage compared to RH, although microvilli remained significantly shorter than those in the Ctrl (*p* < 0.05). The CH group demonstrated substantial toxicological mitigation relative to RH, with no detectable nuclear damage, partial microvilli elongation, and reduced cytoplasmic vacuolation density. No significant pathological changes were detected in the CL group, suggesting a successful reduction in SCP toxicity. However, the optimization of XFP processing techniques remains imperative to ensure consumable safety.

### 3.5. Gut Microbiota Analysis

#### 3.5.1. Alpha Diversity Analysis

**Figure 7:**
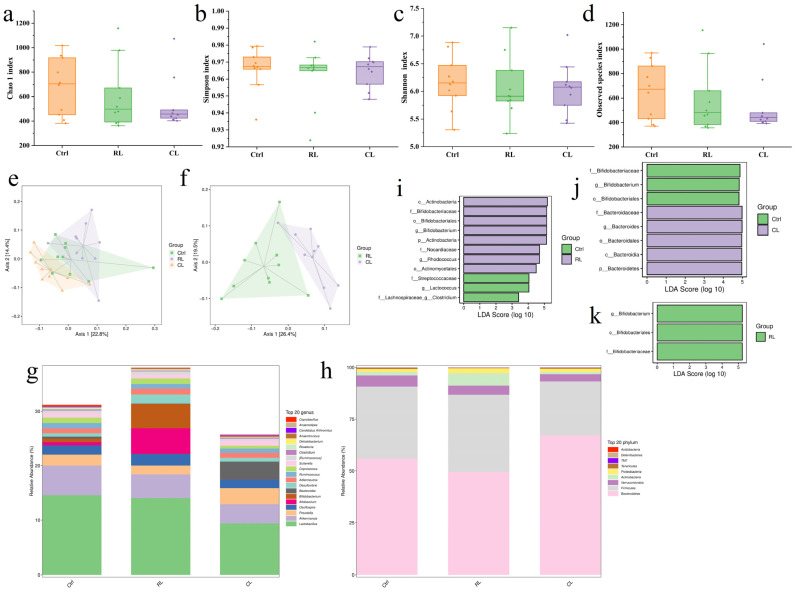
Response of the gut microbiota to SRP and SCP supplementation: (**a**) Chao 1 index analysis; (**b**) observed species index analysis; (**c**) Shannon index analysis; (**d**) Simpson index analysis; (**e**) Beta diversity analysis between Ctrl, RL, and CL groups; (**f**) Beta diversity analysis between RL and CL groups; (**g**) species composition of gut microbiota at the phylum level; (**h**) species composition of gut microbiota at the genus level; (**i**) differential microbial communities between RL and Ctrl groups; (**j**) differential microbial communities between CL and Ctrl groups; (**k**) differential microbial communities between RL, CL, and Ctrl groups.

Alpha diversity indices were applied to evaluate microbial community richness and diversity [85]. Species richness was quantified using the Chao1 and Observed Species indices, while diversity was assessed through Shannon and Simpson indices. As shown in Figure 7a–d, both the RL and CL groups exhibited decreasing trends in gut microbiota richness and diversity, although these changes were not statistically significant (*p* > 0.05).

#### 3.5.2. Beta Diversity Analysis

Beta diversity analysis, performed via principal coordinates analysis (PCoA) [86], revealed compositional variations between the experimental groups. Figure 7e illustrates the Ctrl group’s broad distribution pattern, with partial overlap among all groups. Notably, Figure 7f demonstrates spatial separation between the RL and CL groups, suggesting differential impacts on gut microbiota composition following oral administration.

#### 3.5.3. Microbial Community Composition Analysis

Figure 7g displays the phylum-level composition of murine gut microbiota, dominated by Bacteroidetes, Firmicutes, Verrucomicrobia, Actinobacteria, Proteobacteria, Tenericutes, TM7, Deferribacteres, and Acidobacteria. As shown in Table S2, Firmicutes and Bacteroidetes collectively represent over 90% of the total relative abundance. Compared to the Ctrl group, the RL group exhibited a significant increase in Actinobacteria abundance. Actinobacteria comprise several genera, such as Actinomyces, Streptomyces, Nocardia, and Actinoplanes. Notably, certain species within this phylum are pathogenic; for example, Nocardia can cause nocardiosis [87]. In contrast, the CL group showed a marked elevation in Bacteroidetes, a beneficial bacterial phylum essential for organic matter decomposition, intestinal homeostasis, short-chain fatty acid synthesis, and immune modulation.

At the genus level (Figure 7h), the gut microbiota primarily included Lactobacillus, Akkermansia, Prevotella, and Oscillospira. Table S3 summarizes genus-level alterations in the RL and CL groups relative to Ctrl. A statistically significant increase in Bifidobacterium abundance was observed in the RL group (*p* < 0.05), whereas no significant genus-level changes occurred in the CL group. These results indicate that SRP influenced specific bacterial genera, while SCP maintained gut microbiota stability.

#### 3.5.4. Differential Microbial Community Analysis

LEfSe, a statistical method specifically designed for microbiome studies, identifies species exhibiting statistically significant differences between experimental groups [88,89,90]. Figure 7i reveals distinct bacterial community profiles between the RL and Ctrl groups. Notably, Bifidobacteriaceae [91], Nocardiaceae, Bifidobacterium, and Rhodococcus [92] were significantly upregulated, whereas Streptococcaceae, Lachnospiraceae, Lactococcus, and Clostridium showed marked downregulation. The beneficial genus Bifidobacterium may facilitate purine metabolism [93], potentially reducing systemic uric acid levels. Rhodococcus, capable of degrading complex organic compounds, likely thrived due to residual SRP components reaching the colon, which could serve as carbon/nitrogen sources. This incomplete SRP digestion might also elevate colonic ammonia concentrations, inhibiting ammonia-sensitive Clostridium species. Reduced Clostridium abundance may impair intestinal barrier integrity by diminishing butyrate production. Figure 7j highlights the differential flora between the CL and Ctrl groups, demonstrating increased Bacteroides and reduced Bifidobacterium. Comparative analysis across the Ctrl, RL, and CL groups (Figure 7k) indicates significant Bifidobacterium upregulation exclusively in RL, aligning with Li’s report on plant protein consumption [94]. SRP exerted broader gut microbiota perturbations compared to SCP, partially disrupting microbial equilibrium. Given the TEM-observed structural changes in the small intestine, the further optimization of XF processing is essential to ensure XFP’s safety as a food protein source.

### 3.6. Untargeted Metabolomics and Chemometrics Analysis

Untargeted metabolomics is a scientific approach that provides a comprehensive profile of all the small molecules (known as metabolites) present in a biological sample. Unlike targeted methods, this technique measures all detectable compounds, generating a large and complex dataset [95]. Figure S3 displays the chromatograms of the RL, CL, and Ctrl groups, which show that compounds began eluting at a retention time of approximately 0.6 min with well-resolved peaks. This observation suggests that the use of acetonitrile containing 0.1% formic acid did not affect the chromatographic profiles of early-eluting compounds. The abundance of metabolites identified through untargeted measurements offered valuable insights into the differential effects of raw and concocted XFP on mouse plasma metabolism.

**Figure 8:**
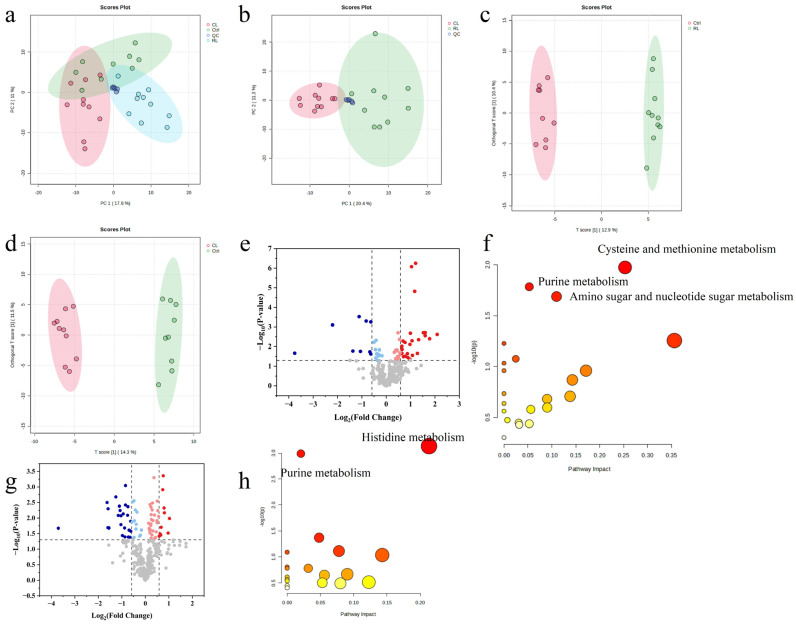
PCA score plot, OPLS-DA score plot, and pathway analysis of various paired experimental conditions: (**a**) PCA score plot between RL, CL, and Ctrl groups; (**b**) PCA score plot between RL and CL groups; (**c**) OPLS-DA score plot between RL and Ctrl groups; (**d**) OPLS-DA score plot between CL and Ctrl groups; (**e**) volcano plot of differential metabolites between RL and Ctrl groups; (**f**) volcano plot of differential metabolites between CL and Ctrl groups; (**g**) pathway analysis between RL and Ctrl groups; (**h**) pathway analysis between CL and Ctrl groups. In subfigures e and g the vertical dashed lines on the “Log2(Fold Change)” axis indicate thresholds for significant fold changes, and the horizontal dashed line near y = 1.3 on the “-Log10(*p*-value)” axis represents the statistical significance threshold. In subfigures (**e**,**g**), data points are colored blue, red, or gray to denote significance and direction of change: blue for significantly down-regulated, red for significantly up-regulated, and gray for non-significant points. In subplots (**f**,**h**), the bar colors represent metabolic pathways, and the red circles represent differential metabolic pathways.

To analyze the extensive data generated by untargeted metabolomics, multivariate chemometric analysis was employed [96]. Figure 8a,b illustrate the separation between experimental groups based on principal component analysis (PCA) [97]. PCA reduces data complexity by identifying principal variations among samples. The QC group samples clustered closely, demonstrating robust experimental quality control. Furthermore, the clear separation between RL and CL groups indicates distinct metabolic impacts of raw and concocted XF proteins on murine plasma.

Orthogonal partial least squares-discriminant analysis (OPLS-DA) identifies systematic differences between defined experimental groups by isolating specific metabolite variations. This method employs statistical validation metrics (Q^2^ and R^2^Y) to evaluate model reliability. OPLS-DA was applied to investigate the effects of RL and CL treatments on murine plasma metabolites. Figure 8c displays the OPLS-DA score plot comparing RL and Ctrl groups (Q^2^ = 0.709, R^2^Y = 0.993), demonstrating robust model validity. The corresponding CL vs. Ctrl comparison (Figure 8d) yielded Q^2^ = 0.502 and R^2^Y = 0.987. Using a threshold criteria of Fold Change (FC) > 1.5, RL/Ctrl ratio, and *p* < 0.05, a total of 35 differential metabolites were identified (Figure 8e). Subsequent pathway analysis via MetaboAnalyst 6.0 revealed 22 perturbed pathways in the RL group (Figure 8f), with three showing statistical significance (*p* < 0.05): cysteine–methionine metabolism, purine metabolism, and amino sugar–nucleotide sugar metabolism. For the CL group, 51 differential metabolites were detected under identical thresholds (Figure 8g). Pathway analysis identified 19 associated pathways, with histidine metabolism and purine metabolism reaching statistical significance (*p* < 0.05, Figure 8h).

A high-protein diet elevates methionine intake, promoting the synthesis of S-adenosylmethionine (SAM) via a methionine adenosyltransferase (MAT)-catalyzed conjugation of methionine and ATP. Under conditions of SAM overaccumulation, metabolic homeostasis is maintained through SAM hydrolysis into S-adenosylhomocysteine (SAH). SAH is subsequently hydrolyzed to homocysteine (Hcy), which enters the transsulfuration pathway for cysteine production [98]. Elevated L-cystathionine levels—an intermediate metabolite in methionine-to-cysteine conversion—reflect augmented transsulfuration activity. This metabolic shift potentiates antioxidant defenses through enhanced glutathione (GSH) biosynthesis [99,100]. Both L-cystathionine and SAM, as pivotal regulators in methionine–cysteine metabolism, demonstrate significant upregulation in the RL/Ctrl group. The disruption of the SAM/SAH ratio serves as a biomarker for diminished detoxification capacity, correlating with increased susceptibility to cardiovascular disorders, neoplasms, and neurodegenerative conditions [101,102]. The concurrent downregulation of D-ribose-1-phosphate, hypoxanthine, and inosine impairs purine metabolism. Specifically, reduced D-ribose-1-phosphate availability constrains ATP generation through impaired purine salvage synthesis, potentially compromising both energy metabolism and T cell priming-mediated immune responses [103]. Depressed hypoxanthine and inosine levels indicate xanthine oxidase inhibition, which may attenuate uric acid production. Agglutinins represent carbohydrate-specific lectins. Although the structural characterization of XF-derived agglutinins remains incomplete, legume-abundant D-mannose-binding proteins undergo intestinal microbiota-mediated degradation, releasing bioactive D-mannose that elevates plasma concentrations [104]. The significant increase in D-mannose content observed in the RL/Ctrl group suggests that agglutinins from raw XF may interfere with amino sugar and nucleotide sugar metabolism through a similar mechanism. As shown in Table S4, these metabolic alterations were associated with systemic imbalances, including the following: suppressed energy metabolism (evidenced by TCA cycle inhibition), dysregulated amino acid homeostasis (methionine/lysine pathway abnormalities), elevated oxidative stress (indicated by lipid peroxidation), and impaired detoxification capacity (demonstrated by SAM/SAH ratio imbalance). KEGG pathway analysis of CL/Ctrl group metabolites (Table S5) revealed no significant enrichment in either glutathione S-transferase-mediated detoxification pathways or glycoconjugate biosynthesis networks. Notably, the CL group exhibited reduced urocanic acid (UA) levels compared to controls. Since UA serves as a key intermediate in histidine metabolism via histidine ammonia-lyase (HAL), this decrease may reflect either HAL enzymatic inhibition or metabolic flux blockage from histidine to UA, ultimately leading to diminished histidine consumption [105].

## 4. Conclusions

In this study, proteins from raw XF and concocted XF were extracted using alkali-soluble acid precipitation and salting-out methods. The secondary structures and functional properties of XFP were characterized through spectroscopic analysis and functional assays. The results demonstrated that ammonium sulfate-extracted protein components retained natural bioactivity. The safety evaluation of SRP and SCP via 14-day murine intragastric administration revealed significant hepato-intestinal toxicity in the RH group. This was evidenced by the following: (1) markedly reduced serum transaminase levels compared to the Control group; (2) inflammatory cell infiltration in hepatic tissue; and (3) diminished jejunal microvillus height. 16S rRNA sequencing indicated that raw XF altered both the diversity and abundance of gut microbiota. Notably, the CL group showed no statistically significant differences from the Control group in these parameters (*p* > 0.05), suggesting that processing effectively mitigated toxicity. However, the CH group exhibited residual toxicity, potentially attributable to incomplete agglutinin inactivation under the current processing conditions. To ensure the safe utilization of processed XFP as a food ingredient, the optimization of processing parameters—including the heating rate, peak temperature, duration, and frying frequency—is recommended. Furthermore, while the current safety assessment was based on short-term experiments, comprehensive long-term toxicity studies are warranted to evaluate dose-dependent effects and processing condition variations.

## Figures and Tables

**Table 1:** Amino acid profile of XFP (g/100 g, n = 3).

	SRP	SCP	AARP	AACP
Asp	7.14 ± 0.17	6.07 ± 0.51 *	6.35 ± 0.07	6.85 ± 0.31
Thr	2.60 ± 0.06	2.08 ± 0.01 **	2.10 ± 0.02	2.09 ± 0.09
Glu	27.32 ± 0.41	28.16 ± 0.15 *	15.06 ± 0.20	16.67 ± 0.82 ^#^
Ser	3.40 ± 0.41	3.35 ± 0.03	2.93 ± 0.03	3.11 ± 0.16
Pro	3.27 ± 0.04	3.03 ± 0.05 **	3.15 ± 0.03	3.42 ± 0.17
Gly	4.97 ± 0.03	4.74 ± 0.03 **	3.32 ± 0.05	3.57 ± 0.15
Ala	2.93 ± 0.04	2.21 ± 0.05 **	3.32 ± 0.02	3.57 ± 0.18
Val	4.24 ± 0.08	3.42 ± 0.02 **	3.31 ± 0.05	3.51 ± 0.12
Met	2.30 ± 0.05	2.13 ± 0.10	0.82 ± 0.02	0.98 ± 0.04 ^##^
Ile	3.47 ± 0.08	2.84 ± 0.04 **	2.82 ± 0.08	3.09 ± 0.17
Leu	6.19 ± 0.12	5.37 ± 0.05 **	4.06 ± 0.06	4.27 ± 0.15
Tyr	1.93 ± 0.03	1.38 ± 0.00 **	1.87 ± 0.02	1.91 ± 0.07
Phe	3.18 ± 0.08	2.61 ± 0.02 **	3.47 ± 0.06	3.77 ± 0.19
Lys	3.91 ± 0.02	2.91 ± 0.04 **	1.71 ± 0.02	1.51 ± 0.08 ^#^
His	2.10 ± 0.04	1.92 ± 0.03 **	1.68 ± 0.02	1.78 ± 0.06 ^#^
Arg	8.90 ± 0.04	8.59 ± 0.02 **	6.21 ± 0.12	6.66 ± 0.28
Cys	3.57 ± 0.04	3.66 ± 0.04 *	0.16 ± 0.00	0.20 ± 0.01 ^##^
Trp	0.61 ± 0.01	0.34 ± 0.01 **	1.00 ± 0.03	1.08 ± 0.02 ^##^
All	92.01 ± 0.90	84.80 ± 0.18 **	65.34 ± 1.49	70.05 ± 3.25

* *p* < 0.05 vs. SRP group; ** *p* < 0.01 vs. SRP group; ^#^ *p* < 0.05 vs. AARP group; ^##^ *p* < 0.01 vs. AARP group. Values are expressed as mean ± SD.

**Table 2:** Organ indices of mice following administration of RH, RL, CH, and CL (mean ± SD, n = 15).

Organ Index	Control	RH	RL	CH	CL
Liver (g per 100 g BW)	4.57 ± 0.68	4.81 ± 0.31	4.72 ± 0.38	4.55 ± 0.22	4.35 ± 0.19
Kidney (g per 100 g BW)	1.86 ± 0.28	1.91 ± 0.20	1.81 ± 0.16	1.81 ± 0.16	1.80 ± 0.08

## Data Availability

Data will be made available on request.

## Supplementary Materials

The following supporting information can be downloaded at: https://www.mdpi.com/article/10.3390/foods14111913/s1. Figure S1: Mice weight gain curve. (Mean ± SD, n = 15); Figure S2: Intestinal content and blood indicators of mice: (a) Aminopeptidase activity in mice; (b) Trypsin activity in mice; (c) Plasma ALT level in mice; (d) Plasma AST level in mice. (n > 13); Table S1: The lengths of the microvilli and the widths of the intercellular connections; Table S2: The top 5 species composition of gut microbiota in mice from Ctrl, RL, and CL groups at the phylum level (n = 10); Table S3: The top 5 species composition of gut microbiota in mice from Ctrl, RL, and CL groups at the genus level (n = 10); Table S4: Differential metabolite changes in RL group/Ctrl group; Table S5: Differential metabolite changes in CL group/Ctrl group.
