# Supplementary material for: Evaluation of the Functional Properties and Edible Safety of Concocted Xanthii Fructus Protein
Source: Foods. 2025 May 28;14(11):1913. doi: 10.3390/foods14111913 (PMC12154445; doi:10.3390/foods14111913)
Supplement: Supplementary file 1 [file foods-14-01913-s001.zip › foods-3591545-supplementary.pdf]

Table S1 The lengths of the microvilli and the widths of the intercellular connections

	microvilli	intercellular connections
Ctrl	956.69±56.65	28.20±2.59
RH	498.8±36.94**	59.25±15.13**
RL	606.2±29.37**	30.67±6.43
CH	881.8±26.35*	34.67±2.31*
CL	968.86±60.17	21.60±4.10*

Values are expressed as mean ± SD. *p<0.05 vs Ctrl group; **p<0.01 vs Ctrl group.

Table S2 The top 5 species composition of gut microbiota in mice from Ctrl, RL, and CL groups at the phylum level (n=10)

Taxon	Ctrl	RL	CL
Bacteroidetes	55.91	49.54	67.29
Firmicutes	34.82	37.30	25.94
Verrucomicrobia	5.43	4.36	3.53
Actinobacteria	1.61	5.99	0.94
Proteobacteria	1.47	2.40	1.65

Table S3 The top 5 species composition of gut microbiota in mice from Ctrl, RL, and CL groups at the genus level (n=10)

Taxon	Ctrl	RL	CL
Lactobacillus	14.60	14.06	9.39
Akkermansia	5.43	4.36	3.53
Prevotella	2.04	1.61	2.95
Oscillospira	1.68	2.17	1.59
Allobaculum	0.61	4.69	0.02
Bifidobacterium	0.63	4.49	0.01

Table S4 Differential metabolite changes in RL group/Ctrl group

Metabolites	log_2(FC)	P	trend
2-Furoic acid	1.27	0	↑
Citric acid	1.1	0	↑
Malonic acid	1.22	0	↑
Methanesulfonic acid	-1.05	0	↓
D-Ribose-1-phosphate	-2.19	0	↓
3-Hydroxy-3-(methoxycarbonyl)pentanedioic acid	-0.78	0	↓
5-Hydroxymethyl-2-furaldehyde	1.63	0	↑
Prostaglandin K2	1.59	0	↑
9-Oxo-ODE	1.66	0	↑
L-Cystathionine	0.6	0	↑
8-Isoprostaglandin E2	2.14	0	↑
Bioresmethrin	1.83	0	↑
Phenylacetaldehyde	1.12	0	↑
D-Mannose	1.04	0	↑
1-Vinylimidazole	0.74	0.01	↑
Imazapic	1.04	0.01	↑
S-Adenosyl-methionine	0.62	0.01	↑
N-Acetyl-D-galactosamine 4-sulfate	-0.65	0.01	↓
L-Iditol	0.7	0.01	↑
L-Pyroglutamic acid	0.69	0.01	↑
D-Galactose	0.62	0.01	↑
Diethanolamine	0.71	0.01	↑
Hypoxanthine	-1.36	0.02	↓
L-Saccharopine	0.92	0.02	↑
N-Glycolylneuraminic acid	0.63	0.02	↑
3-Sulfinoalanine	1.32	0.02	↑

1-Methylnicotinamide	0.82	0.02	↑
N-Acetyl-DL-glutamic acid	-1.03	0.02	↓
Ipratropium	0.94	0.03	↑
Bilirubin	-3.72	0.03	↓
4-Guanidinobutyric acid	0.74	0.03	↑
Carnosine	0.59	0.04	↑
3-(2,6-Dioxocyclohexyl)propanenitrile	1.03	0.04	↑
L-Threonine	0.81	0.05	↑
Inosine	-1.46	0.05	↓

Table S5 Differential metabolite changes in CL group/Ctrl group

Metabolites	$\log_2$(FC)	P	trend
Trimethylamine N-oxide	-0.931	0	↓
Xanthurenic acid	-1.732	0	↓
Indole-3-carboxylic acid	-1.686	0	↓
Phosphoric acid	-1.259	0	↓
4-Aminobenzoic acid	-1.352	0	↓
2'-Deoxyuridine	-0.615	0	↓
2-Furoic acid	0.686	0	↑
Urocanic acid	-0.837	0	↓
N-Acetyl-DL-glutamic acid	-1.129	0	↓
1-Methyladenine	-1.661	0	↓
Nipecotic acid	-0.848	0	↓
Cyclohexanecarboxylic acid	-0.657	0	↓
1-Vinylimidazole	-0.943	0	↓
Hippuric acid	-1.689	0	↓
Tiglic acid	-1.219	0	↓
Uric acid	-1.04	0	↓

Citric acid	0.655	0.01	↑
Trigonelline	-0.718	0.01	↓
2,4-Dihydroxybenzoic acid	-1.139	0.01	↓
2,4-Quinolinediol	-0.915	0.01	↓
3,4-Dihydroxybenzenesulfonic acid	-1.082	0.01	↓
Bilirubin	-3.839	0.01	↓
Carnosine	0.732	0.01	↑
Adenosine	0.939	0.01	↑
Catechol	-0.968	0.01	↓
D-Arabinose	-0.721	0.01	↓
2'-O-Methyluridine	-0.991	0.01	↓
Succinic semialdehyde	-1.182	0.01	↓
3-Methylhistamine	0.935	0.01	↑
Xanthosine	1.436	0.01	↑
Adenine	0.632	0.02	↑
6-Aminocaproic acid	-0.795	0.02	↓
4-Guanidinobutyric acid	-0.907	0.02	↓
5-Hydroxyindole	-0.919	0.02	↓
D-Fucose	-0.619	0.02	↓
Methanesulfonic acid	-0.6	0.02	↓
Azelaic acid	-0.616	0.02	↓
8-Isoprostaglandin E2	1.662	0.03	↑
N-Isobutyrylglycine	-0.809	0.03	↓
Imidazoleacetic acid	-0.643	0.03	↓
9-Oxo-ODE	1.116	0.03	↑
Phenylacetylglycine	-0.752	0.04	↓
Phenylacetaldehyde	0.738	0.04	↑
Bioresmethrin	1.661	0.04	↑

3-Hydroxypyridine	-1.673	0.04	↓
3-(2,6-Dioxocyclohexyl)propanenitrile	-1.048	0.04	↓
N-Acetyl-L-tyrosine	1.412	0.04	↑
2-Ketoadipic acid	-1.046	0.05	↓
Ipratropium	1.131	0.05	↑
Glutaric anhydride	-0.795	0.05	↓
Uridine	0.592	0.05	↑

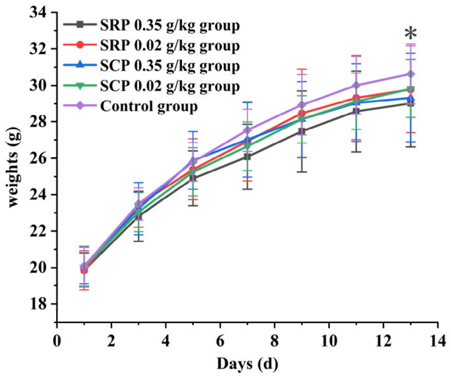

Fig. S1. Mice weight gain curve. (Mean±SD, n=15)

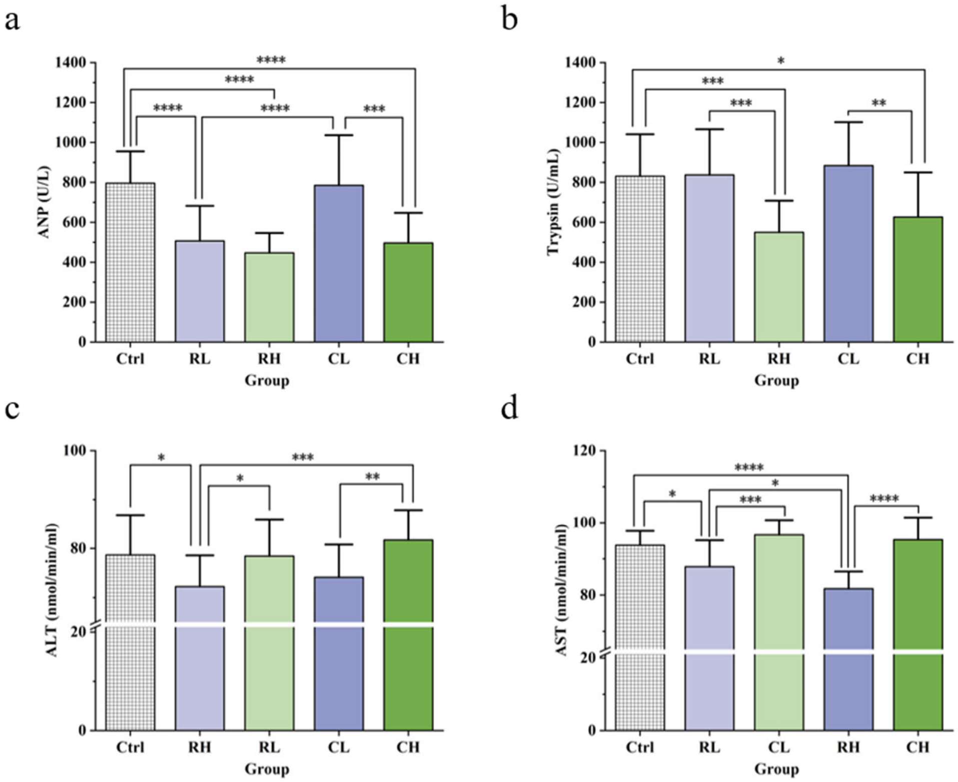

Fig. S2. Intestinal content and blood indicators of mice: (a) Aminopeptidase activity in mice; (b) Trypsin activity in mice; (c) Plasma ALT level in mice; (d) Plasma AST level in mice.) (n>13)

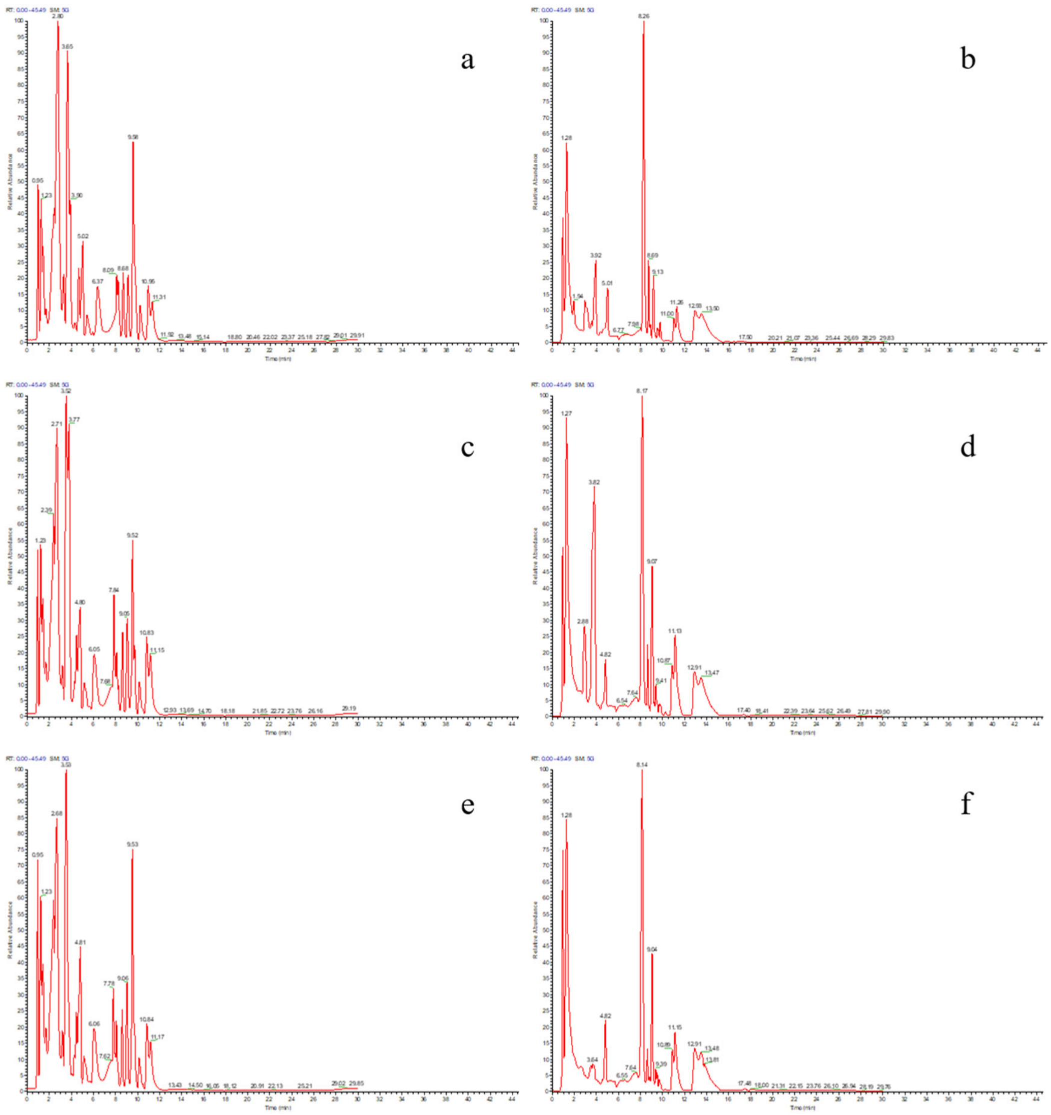

Fig. S3. Representative total ion chromatograms (TIC) of the (a) RL group and (c) CL group and (e) Ctrl group based on positive ion mode. Representative TIC of the (b) RL group and (d) CL group and (f) Ctrl group based on negative ion mode.
